# Supplementary material for: An NMDAR positive and negative allosteric modulator series share a binding site and are interconverted by methyl groups
Source: eLife. 2018 May 24;7:e34711. doi: 10.7554/eLife.34711 (PMC5967867; doi:10.7554/eLife.34711)
Supplement: Supplementary file 1. [file elife-34711-supp1.docx]

**Supplemental Table S1.** Comparison of **EU1794-2** effects on NMDAR responses to saturating and sub-saturating concentrations of agonist

|  | **EC_50_ (µM) [conf. int.]^a^**  **Maximal Degree of Modulation (% of control)^b^** | | | |
| --- | --- | --- | --- | --- |
| **EU1794-2** | **GluN2A** | **GluN2B** | **GluN2C** | **GluN2D** |
| **Saturating agonist** | 0.60 [0.44, 0.82]^†^  6 ± 2% | 1.2 [0.8, 1.9] ^†^  10 ± 3% | 0.21 [0.18, 0.25]^†^  15 ± 1% | 0.20 [0.17, 0.25]^†^  14 ± 1% |
| **Sub-Saturating agonist** | 1.8 [1.1, 3.0]  37 ± 7% | 1.4 [1.1, 1.7]  28 ± 1% | 0.72 [0.52, 1.0]  41 ± 3% | 0.32 [0.26, 0.40]  24 ± 3% |

*^a^* EC_50_ values were obtained by least-squares fitting of data from individual experiments by the Hill equation. EC_50_ values are given as the mean with the 95% confidence interval determined from log(EC_50_). Sub-saturating agonist concentrations were 0.6 µM glutamate and 0.2 µM glycine.

*^b^* The extent of modulation is given as a percent of the control response in the absence of test compound. The maximal degree of modulation is mean ± SEM. Sub-saturating agonist data are from 6 oocytes.

^†^ Data from Table 1 is included here for clarity.
