# Supplementary material for: An NMDAR positive and negative allosteric modulator series share a binding site and are interconverted by methyl groups
Source: eLife. 2018 May 24;7:e34711. doi: 10.7554/eLife.34711 (PMC5967867; doi:10.7554/eLife.34711)
Supplement: Supplementary file 2. [file elife-34711-supp2.docx]

**Table S2.** Enantiomeric preference of **EU1794-4**.

|  | **IC_50_ (µM) [conf. int.]^a^**  **Maximal Modulation Extent (% of control)** | | |  |
| --- | --- | --- | --- | --- |
| **Compound** | **EU1794-4** | **(-)-EU1794-4** | **(+)-EU1794-4** |  |
| **GluN2A** | 2.2 [1.8, 2.8]^†^  32 ± 3% | 4.9 [2.1, 12]  17 ± 10% | 12 [7.4, 20]  41 ± 6% |  |
| **GluN2B** | 2.6 [1.4, 4.8] ^†^  67 ± 4% | - | - |  |
| **GluN2C** | 0.42 [0.28, 0.61] ^†^  52 ± 2% | 0.48 [0.27, 0.86]  47 ± 1% | 1.7 [0.99, 3.0]  50 ± 4% | * |
| **GluN2D** | 0.36 [0.29, 0.45] ^†^  51 ± 3% | 0.46 [0.10, 1.1]  54 ± 3% | 3.5 [1.6, 7.5]  47 ± 6% | * |

*^a^* EC_50_ values were obtained by least-squares fitting of data from individual experiments by the Hill equation. EC_50_ values are given as the mean with the 95% confidence interval determined from log(IC_50_); the maximal degree of modulation is given as mean ± SEM. Data are from 4-5 oocytes from 2 independent experiments. Data were not fitted (shown as -) if the response recorded at 30 µM of test compound did not differ by more than 15% from control.

† Data from Table 1 is included here for clarity.

^*^ indicates non-overlapping IC_50_ confidence intervals of **(-)-EU1794-4** and **(+)-EU1794-4**.
